# Dairy cows did not rely on social learning mechanisms when solving a spatial detour task

**DOI:** 10.3389/fvets.2022.956559

**Published:** 2022-09-07

**Authors:** Johanna Stenfelt, Jenny Yngvesson, Harry J. Blokhuis, Maria Vilain Rørvang

**Affiliations:** ^1^Department of Biosystems and Technology, Swedish University of Agricultural Sciences, Lomma, Sweden; ^2^Department of Animal Environment and Health, Swedish University of Agricultural Sciences, Skara, Sweden

**Keywords:** cognitive task, cattle, animal welfare, social transmission, animal learning, cognition, bovine, observational learning

## Abstract

As herd-living animals, cattle have opportunities to observe and learn from others. While there is evidence of simpler processes of information transfer in cattle (social facilitation and stimulus enhancement), true social learning mechanisms in cattle remain largely unexplored. This study aimed to investigate if dairy cows possess cognitive abilities to acquire new behavior through social learning in a spatial detour task. Thirty-two dairy cows (ages 2–9 years) participated in the study. A food reward was placed behind a U-shaped formation (4 x 2 m), allowing the cows to see but not reach the reward without first detouring around the obstacle. The U-shape provided two routes (~18 m walking distance) to the reward, of which one was used for demonstration. Two cows were demonstrators and 30 cows were divided into two groups, assigned as either observers of demonstration (*n* = 15) or controls not observing demonstration (*n* = 15). Cows had three attempts (trials) to solve the task. Response variables were: success, latency to reach the reward, concordance in choice of route to detour, and time spent facing the test arena before each trial started. The study found no significant differences in success or latency between observers and controls, although observers spent a greater proportion of the time before trials facing the test arena. However, successful observers tended to be faster than successful controls. Individual cows were generally consistent in their choice of route, and cows choosing the demonstrated route were significantly faster than cows that did not. Success in solving the task decreased over trials, likely due to decreasing food motivation. Age had a significant effect on success in 2^nd^ and 3^rd^ trial, with younger cows being more successful. The lacking effect of treatment on success suggests that the age effect may be explained by a higher motivation, rather than social learning. Adding to the sparse knowledge of social learning in farm animals, these results indicate that cows did not utilize social learning mechanisms when solving the detour task. Future research should focus on clarifying whether cattle possess cognitive abilities necessary for social learning, as well as if /when social learning is a primary strategy.

## Introduction

Animals can acquire new behavior through individual and social learning. Individual learning can occur through an individual's own experience, e.g., of trial and error, whereas social learning is influenced by observing or interacting with others (1, 2). In an unpredictable environment where the consequences of failing through trial and error may be dire, learning by observing others can be a beneficial strategy to acquire new information at reduced costs (1). Observing others may also facilitate individual learning in situations where an animal's behavior is influenced by the observation but the actual learning is a direct result of the animal's own experience, rather than the observation itself. For example, synchronized behaviors rely on social facilitation where the motivation to perform an already established behavior is increased after observing other individuals performing that same behavior (3). Social facilitation can thus be considered a social influence on behavior, not a form of learning, as it only leads to an increase (or decrease) in the performance of an existing behavior (3, 4). Through stimulus enhancement, observing other individuals interact with a specific stimulus (e.g., a novel drinker) can increase the motivation of an animal to investigate that same stimulus, subsequently creating an opportunity for individual associative learning of how to operate it (4, 5). These processes of information transfer that facilitate individual learning are collectively referred to as social transmission and differ from true social learning (6).

Evidence of social learning in cattle is scarce. Previous studies have focused on the transmission of information from parent to offspring, or from older to younger individuals. For example, calves develop preferences for pasture locations and habitats based on early-life experiences of grazing together with their dam or foster dam (7), and naïve heifers are quicker to start grazing when grouped with older, pasture-experienced cows compared to when grouped with naïve peers (8). These findings can, however, be explained by simpler processes of information transfer such as stimulus enhancement and social facilitation (i.e., social transmission mechanisms), and are thus not evidence of true social learning. In studies on sheep, another grazing livestock, lambs have been shown to learn which food to eat and which food to avoid from grazing with their dams [e.g., (9, 10)]. Such observations are indicative of social learning mechanisms if the lamb (or calf) learns to eat or avoid a novel food resource, which it has no prior experience with, and expresses this behavior without the parent present. Social influences (regardless of the cognitive mechanisms involved) on feed intake, feed selection as well as the sampling of novel, potentially toxic food decrease the risks associated with trial-and-error learning in foraging (11).

Other studies have investigated the transmission of information between peers. Heifers presented with an operant task of pushing a panel to access a food reward do not improve in learning the task after observing it performed but spend longer time engaging in the task if they first observe a demonstration (12), hence a clear example of stimulus enhancement. Naïve heifers will be more successful in finding feed locations in a maze when accompanied by a trained peer (13). Cows can be socially influenced by the response of other cows when determining what distance to keep from an aversive handler (14). Likewise, responses to virtual fences can also be socially facilitated, with cattle staying within the intended zones based on the response of peers to auditory and electrical cues (15). Recently, Stenfelt et al. (16) found that a calm companion lowered fear in small groups of dairy cows (*n* = 4) when exposed to the novel and aversive stimulus of the opening and closing of an umbrella. Like the findings of transmission of information between cattle parent and offspring, these findings of transmission between cattle peers can be explained by the simpler processes of stimulus enhancement and social facilitation (i.e., social transmission mechanisms). As mentioned, a social influence on the performance of an existing behavior can be distinguished from the learning of a new behavior (3, 4). Hence, more research is needed to establish whether cattle have, and make use of, the ability for true social learning when acquiring new behavior from conspecifics, whether parents or peers.

Social learning requires cognitive abilities of higher complexity than social transmission (6, 17, 18). Thus, the copying of an individual's motor pattern, also referred to as imitation, requires the observing animal to match the visual representation of the demonstrator with its own proprioceptive control (6, 18). Reproducing the results of an individual's behavior rather than the precise behavior itself, also referred to as goal emulation, requires the observer to make a connection between the insights gained from observing and the observer's own motivations (6, 19). Solving a spatial detour task is a method previously used to investigate social learning in animals [e.g., (20–25)]. In a detour task, the animal must navigate around an obstacle to reach a certain goal, e.g., a reward. This requires momentarily moving away from the goal, i.e., increasing the distance to the reward, in order to reach it. For gregarious ungulates, like cattle, who in their natural environment would navigate over large distances and through changing terrains, a spatial task seems to be of greater biological relevance than, e.g., an operant task which is not a part of their natural environment. Hence, exploring social learning in this spatial context would give valuable information about the cognition of cattle.

The information transfer between conspecifics (e.g., cow to cow), as well as heterospecifics (e.g., human to cow), can be studied by allowing an animal to observe a trained demonstrator performing the spatial detour task. This has not yet been used in social learning experiments with cattle, but with several other species and with varying results. For example, domestic dogs (*Canis lupus familiaris*) have been shown to use inter-species social learning when solving a detour task after observing a human demonstrator, although they did not copy the demonstrator's exact route (20). Sanctuary-raised dingoes (*Canis lupus dingo*) were tried in an equal experiment and proved more successful than domestic dogs in solving the detour task, although their performance was unaffected by a human demonstrator (21). The lack of inter-species social learning in dingoes indicates that the ability of dogs to learn from human demonstrators in a detour task may be a result of the increased attentiveness to, and ability to read, human communicative signals following the domestication process (26). Being a domesticated species, it is likely that cattle too, at least to some extent, have been selected to pay attention to human body language.

Like domestic dogs, domestic goats (*Capra aegagrus hircus*) appear to use inter-species social learning, as they were significantly helped in solving the detour task by observing a human demonstrator (23). This is in contrast with the results of studies on domestic horses (*Equus caballus*), which have not been shown to benefit from demonstrations from humans or conspecifics (22, 24). The presence of a conspecific behind the obstacle has, however, shown to impact the detour strategy of horses (25), potentially indicating that social companionship (or lack thereof) may be an important aspect to consider when designing a detour task. As the cognitive mechanisms of domesticated ungulates appear to vary between species, it is possible that cattle may possess the ability to learn from observing others. Learning more about the social cognition of cattle will help us in our understanding of their social environment and provide insight into how cattle acquire new knowledge and behavior. This study aimed to investigate if dairy cows possess the cognitive abilities to acquire new behavior through intra-species social learning in a spatial detour task. The main hypothesis was that cows observing a trained demonstrator cow performing the detour task would be more successful in solving the task and do so with shorter latencies compared to control cows that did not observe such demonstration.

## Materials and methods

### Ethical considerations

The details of the experiment were assessed and approved by the “Board for Animals in Research and Teaching” at SLU, Sweden. Of the three experimenters who took part in the training and testing, two had an education in responsible use and treatment of animals used in research and supervised the third experimenter (MSc student). All methods used and care for the animals complied with national legislation on animal experimentation by the Swedish Board of Agriculture (27) and met the ARRIVE guidelines (28) as well as complied with the ethical guidelines proposed by the Ethical Committee of the ISAE (International Society of Applied Ethology) (29). The director of Uddetorp Agricultural School and the staff involved were informed about, and agreed to, the details of the study.

### Animals and housing

Thirty-two dairy cows of Uddetorp Agricultural School in Skara, Sweden, participated in this study. The training of cows and data collection took place over the course of 3 weeks in June of 2021. During this time, the cows were loose housed in a free-stall cowshed, with at least 12 h of pasture access per day. In addition to grass from being pastured, cows were fed a partial mixed ration with concentrates in transponder-controlled feeders and had *ad libitum* access to water. The cows were a mixture of Swedish Holstein (*n* = 20) and Swedish Red (*n* = 12), with the uneven distribution between breeds due to availability on the farm. The cows were of varying age (2–9 years), in varying parity (1^st^-6^th^ parity), and in various stages of the lactation cycle. Cows that had recently calved were given at least 1 week to recuperate before joining the experiment, and cows expected to calve within a week from the day of testing were excluded.

#### Demonstrators, observers and control cows

The dominance relationship between demonstrator and observer has been suggested to be important for the facilitation of social learning (30), with dominant animals making better demonstrators (1, 22). In previous studies on horses, demonstrators have been selected based on the results of investigations into the dominance hierarchy (22, 25). Such an investigation was not feasible within this study, instead, the demonstrators were chosen based on brief behavioral observations during interactions with herd members. On three separate occasions, the experimenters visited the herd on pasture as well as in the cowshed and assessed (i) the success of initiating movement of one or more followers, (ii) the willingness of the cows to approach and interact with the handlers, and (iii) winning agonistic encounters between herd members. When conducting the first two observations, the experimenters walked around in the pasture and noted which cows voluntarily approached the experimenters. The experimenters further noted which of the approaching cows seemed to attract a following of other cows previously reluctant to approach on their own. Lastly, the experimenters noted agonistic interactions that occurred during this time, e.g., butting (31, 32). When conducting the third observation, the experimenters visited the cows in their free-stall cowshed and again noted which cows would approach, which appeared to initiate the approach of other cows, as well as agonistic interactions. The visits lasted for ~15–20 min. Two cows met the stated requirements and were selected to participate as demonstrators. Both demonstrators were 3 years old, one Swedish Holstein and one Swedish Red.

The remaining 30 cows were divided into two groups, balanced primarily on breed and age, but also with some consideration to brief observations of behavior displayed during the habituation process (see Habituation to experimental venue for details) e.g., agonistic encounters between group members eating from or approaching the same bucket, if a cow seemed shy/fearful of the experimenter refilling and moving buckets around within the experimental venue, and if a cow seemed highly motivated to obtain the food reward or was observed grazing before approaching the refilled buckets. The two groups were then randomly assigned as either observer (*n* = 15) or control (*n* = 15).

### Experimental design

**Figure 1:**
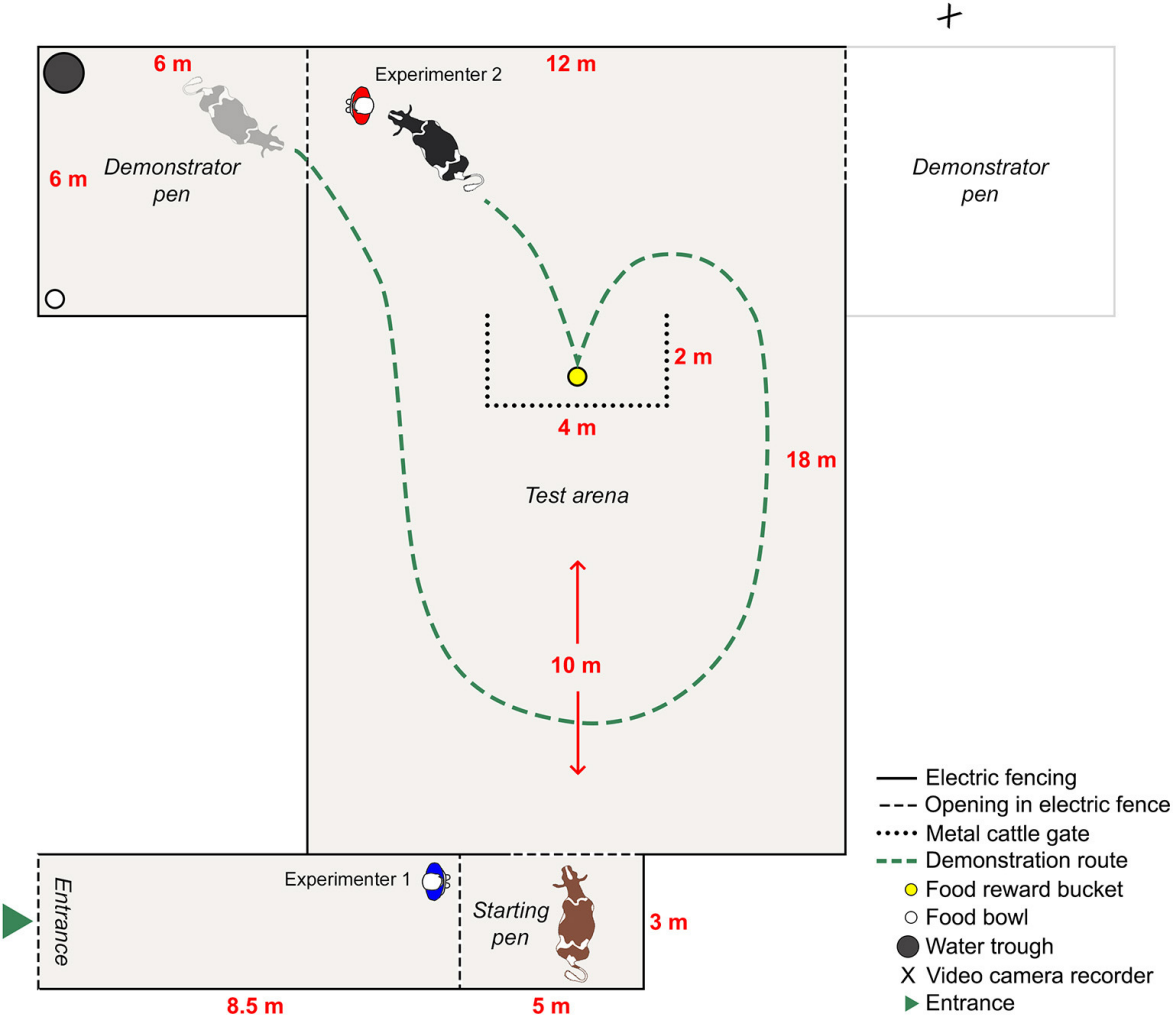
The layout of the experimental venue during a right-sided demonstration of the spatial detour task, where the black cow is the demonstrator and the brown cow the observer. The grayed-out demonstrator pen was used for left-sided demonstrations, during which the video camera was moved to the opposite side of the test arena.

The cows were presented with a yellow bucket that contained a food reward (pelleted concentrates). This specific concentrate was part of the cows' partial mixed ration and was chosen as the food reward per the suggestion of the farm staff, as they knew the cows to be highly motivated to obtain it. The type of bucket (Red Gorilla flexible TubTrug 26 L) and its yellow color were chosen to ensure the reward bucket differed from the black buckets with metal handles typically used at the farm, and to ensure that the cows could differentiate it from the green grass (33). The yellow reward bucket was positioned behind a U-shaped obstacle of metal cattle gates, which allowed the cow to see the bucket but not reach it without first going around the gates (Figure 1), i.e., solving a spatial detour task. The aim was to assess differences in the response (latency) and success rate of completion of the task over three consecutive trials (i.e., three attempts carried out on the same day), between cows in a treatment group that first observed a trained demonstrator cow solve the task and reach the reward bucket, and cows in a control group that did not receive the demonstration.

#### Habituation to experimental venue

The experimental venue consisted of a fenced-off section of the cows' regular pasture. All cows were habituated to the experimental venue and the yellow reward buckets in small, randomly assembled groups of 3-4 cows. The groups were driven into the test arena where four yellow buckets, containing a handful of food each, were randomly distributed across the experimental venue. As the cows finished the content of a bucket, it was instantaneously refilled and moved to a new location within the test arena. Thus, the cows were continuously seeking food from the presented reward buckets. All cows were habituated in this way for 10 min on two separate occasions. The cows were considered habituated if, when released into the test arena alone, they immediately walked up to and ate from the reward bucket in its designated place (without the cattle gates present) during minimum 30 s. Before the detour test took place, all cows were pre-tested once on the habituation criterion individually.

#### Training of demonstrators

The two demonstrator cows were trained to follow one of the experimenters carrying a black bucket containing food (pelleted concentrates), along the demonstration route (Figure 1). The demonstrator was regularly allowed to eat from the black bucket to reinforce her motivation to follow the experimenter. When reaching the yellow reward bucket behind the cattle gates, a handful of food was dropped into the reward bucket and the demonstrator was allowed to eat from it before continuing with a second and third identical lap, before being placed in the demonstrator pen (Figure 1). The demonstrators were trained for ~20 min each on two separate occasions prior to the detour test. The demonstrators were considered trained and habituated if they, during at least three consecutive laps around the test arena, consistently followed the experimenter and could shift their focus to eat from the yellow reward bucket and then back to following the experimenter again.

#### Detour test

The detour test took place on three separate days. The 30 cows were divided into three subsets which were balanced on treatment, and each subset was tested on one of the three test days (day 1: *n* = 10, day 2: *n* = 10, day 3: *n* = 10). All cows were tested within a week of complying with the habituation criterion. The cattle gates were assembled out of sight for all cows, and the demonstrator was placed in its demonstrator pen (either to the left or the right, which was balanced to control for laterality of the cows) with a large tub of water and an empty white food bowl. The test subject (i.e., observer/control) was collected from the cowshed and driven to the test arena by use of negative reinforcement if needed (e.g., gently tapping on the cow, using soft sounds, and/or gesticulating to encourage walking). Extra care was taken to avoid stressing the cows and to make sure they arrived at the test arena as calmly as possible. Observers and controls were alternated throughout the test and were always given either left-sided or right-sided demonstrations, never both. The side of the demonstration (i.e., left/right) was balanced over test days.

#### Test procedure for observers

The observer cow was placed in the starting pen by experimenter 1, who then remained on the left side of the starting pen (Figure 1). The demonstrator cow was led out onto the test arena by experimenter 2 and the demonstration began. As per prior training, the demonstrator followed experimenter 2 and the black bucket along the demonstration route and stopped to eat a handful of food from the yellow reward bucket before continuing with a second and third lap (i.e., all observer cows received three demonstrations before their first trial). After the third lap, the demonstrator was led back to the demonstrator pen, where experimenter 2 dropped a handful of food in the white food bowl to keep the demonstrator cow occupied while securing the arena with electric fence gate handles. Experimenter 2 then walked over to the yellow reward bucket within sight of the observer, dropped a handful of food in the bucket with a rattling sound to ensure the attention of the observer, and walked over to release the observer from the starting pen. Experimenter 2 walked the demonstration route (as to not provide any human demonstration of the opposite route), i.e., behind the cattle gates on the demonstration side and exiting on the side of the demonstrator pen (Figure 1).

To release the observer from the starting pen, experimenter 2 opened the gate and stepped into the starting pen on the cow's right side (opposite of experimenter 1) for a symmetrical pressure of both sides so as to not influence the choice of route. Both experimenters remained at the starting pen during the trial. The latency to reach the reward bucket was measured from the moment both hind legs of the observer were positioned in the test arena until her muzzle reached the bucket. If the observer did not take the demonstration route, or if she failed to reach the bucket within 90 s, the attempt was considered unsuccessful, and the observer was retrieved to the starting pen using the reward bucket as a motivator. When the observer was back in the starting pen, the reward bucket was returned to its designated place and a one-lap demonstration was performed before the observer was released for a new trial. If the observer took the demonstration route and reached the reward bucket within 90 s, the attempt was considered successful. The observer was then retrieved to the starting pen using the reward bucket and released for a new trial as soon as the reward bucket had been returned and refilled. Observer cows were given a total of three consecutive trials (attempts) within the test day.

#### Test procedure for controls

The control cows were placed in the starting pen where they waited 3 min, corresponding to the duration of a three-lap demonstration for observers. During this time, the demonstrator remained in its pen, and experimenter 2 waited outside the demonstrator pen, next to the gate (Figure 1). Before the control was released, experimenter 2 walked to the yellow reward bucket within sight of the control, dropped a handful of food in the bucket with a rattling sound (as to allow for the same stimulus enhancement as for observers), and walked over to release the control from the starting pen. Experimenter 2 walked the same route as a demonstrator cow would have; behind the cattle gates on the demonstration side and exiting on the side of the demonstrator pen. The control was released in the same way as the observer, with one experimenter on each side of the starting pen. After 90 s or upon reaching the reward bucket, regardless of the route taken, the control was retrieved to the starting pen using the reward bucket, where she waited 1 min (corresponding to the time of a one-lap demonstration as to offer controls the same amount of time to observe the test arena and the spatial problem, as the maximum wait between observer trials) before being released into the test arena for the next trial. Control cows were given a total of three consecutive trials (attempts) within the test day.

#### Recording

Demonstrations and trials of both treatment groups were continuously recorded using a video camera on a stationary tripod. The completion of the detour task (yes/no), the chosen route (demonstration/opposite), and the latency from release to completion (s) were recorded on-site and later confirmed by use of the video footage. The video footage was also later used for continuous recording of the time individual cows of both treatment groups spent facing the test arena in the time before each trial started. The time spent facing the test arena was recorded as the total duration of the cow having her head lifted from the ground and facing the test arena/spatial problem at a maximum of ~22.5° away from the center of the test arena. The 22.5° on each side of the center together made up an area of ~45° in total. In practice, this covered the width of the test arena and ~1–1.5 m of both demonstrator pens to where the video camera was located (Figure 1). Concordance in route (demonstration/opposite) was recorded as the number of trials in which the cow repeated the route taken in her first successful attempt, as an indication of a potential learning process. Concordance could vary between 0 (if route in both second and third trial differed from first trial) and 2 (if route in both second and third trial were the same as in first trial).

### Data editing

The sample size for the statistical analysis of success was 30 cows (*n* = 15 observers, 15 controls) and a total of 90 trials (*n* = 45 observers, 45 controls). Latency was recorded for all trials in which the test cow solved the task within 90 s. The sample size for the statistical analysis of latency was thus 29 cows (*n* = 14 observers, 15 controls) and a total of 61 trials (*n* = 32 observers, 29 controls). Some video footage was unfortunately lost due to technical difficulties, thus the sample size for the statistical analysis of time spent facing the test arena was 27 cows (*n* = 13 observers, 14 controls) and 79 trials (*n* = 38 observers, 41 controls). Concordance in route over trials included test cows that reached the bucket during their first attempt and thus had a learning opportunity in first trial with two chances to repeat the success in the following trials. The sample was further standardized by only including cows that passed the obstacle in both second and third trial, and thus had the opportunity to approach the reward bucket if they wanted to. The final sample size for the statistical analysis of concordence in route was thus 20 cows (*n* = 10 observers, 10 controls) and a total of 60 trials (*n* = 30 observers, 30 controls).

### Statistical analysis

All statistical analyses were performed in R version 4.1.0 (34) with RStudio version 1.4.1717 (35), using the packages lme4 (36), emmeans (37), nnet (38), MASS (38), car (39), DHARMa (40), Rmisc (41) and tidyverse (42). *P*-values below 0.05 were considered significant.

#### Success

The success of each cow in each trial was recorded as a categorical variable with three levels: complete success (reaching bucket within 90 s through demonstration route), partial success (reaching bucket within 90 s through opposite route), and no success (failing to reach bucket within 90 s regardless of route). Two generalized linear mixed-effect models (GLMMs) were employed to investigate effects of treatment (categorical variable with two levels: observer/control), trial number (categorical variable with three levels: first/second/third), age (numeric variable, mean ± SD = 3.7 ± 1.5 years) and breed (categorical variable with two levels: Swedish Holstein/Swedish Red), with cow as a random factor. Each model included a binomial response of success (i.e., success vs. no success): the first model considered both partial and complete success (vs. no success), whereas the second model only considered complete success (vs. partial and no success). This yielded two analyses, one on the success of reaching the reward bucket (i.e., solving the detour task regardless of route), and one on the success of doing so through the demonstration route (i.e., solving the detour task using the demonstrated route). This model type was chosen over multinomial models to account for repeated measures on each cow, as each cow was tested in three trials. As the primary response variable was binary (i.e., successful or not), data was analyzed using a logistic regression. Estimated marginal means (EMMs) were calculated for all fixed effects. As initial analyses of success revealed an unexpected decrease in success over trials, a *post hoc* analysis was carried out analyzing first trial separately. For this, a multinomial model was used, including fixed effects of treatment, age and breed. EMMs were calculated and used for pairwise comparisons of all fixed effects.

#### Latency

Latency was defined as the time it took for the cow to reach the yellow reward bucket from the moment both hind legs stepped onto the test arena. To account for the repeated measures on each cow, a linear mixed-effects model (LMM) was employed to investigate fixed effects of treatment, trial number, age (mean ± SD = 3.4 ± 1.6 years), breed and choice of route (categorical variable with two levels: demonstration/opposite), with cow as a random factor. EMMs were calculated for all fixed effects.

#### Facing of test arena

The time spent facing the test arena was considered as a percentage of time before each trial (as this time varied depending on if one-lap, three-lap, or no demonstration was performed) and was analyzed using a LMM considering fixed effects of treatment, trial number, age (mean ± SD = 3.7 ± 1.6 years) and breed, with cow as a random factor. EMMs were calculated and used for pairwise comparisons of all fixed effects.

#### Concordance in route

Concordance in route was defined as the number of times the cow repeated the route taken in her first successful attempt, and summarized as an individual score. The total score (0-2) for each cow was analyzed in an ordinal logistic regression model considering fixed effects of treatment, age (mean ± SD = 3.5 ± 1.8 years) and breed. EMMs were calculated and used for pairwise comparisons of all fixed effects.

## Results

### Success

**Figure 2:**
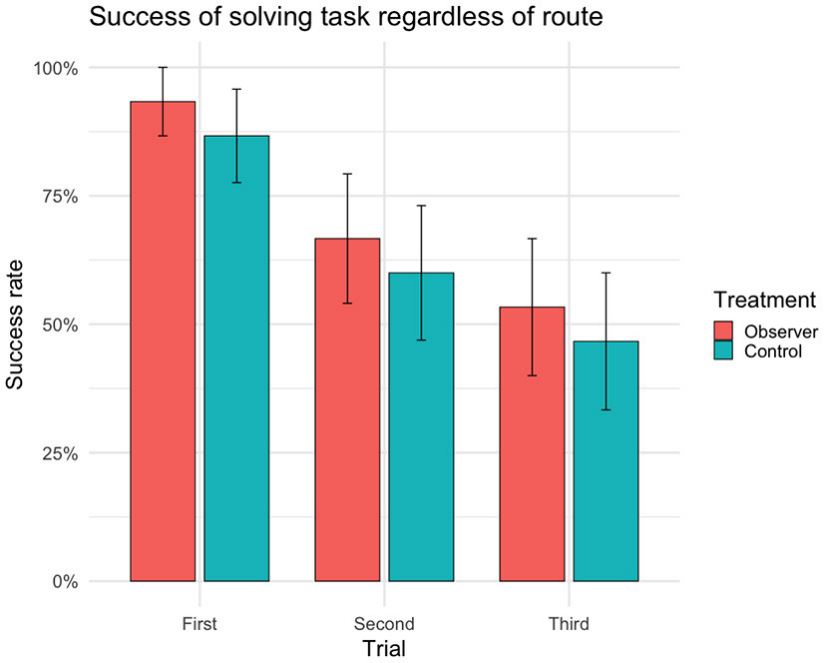
Success rate (sample mean) of reaching the yellow reward bucket and solving task within 90 s regardless of route (i.e. achieving complete or partial success) for each trial (*n* = 30 cows, 90 trials). Error bars indicating SEM.

**Figure 3:**
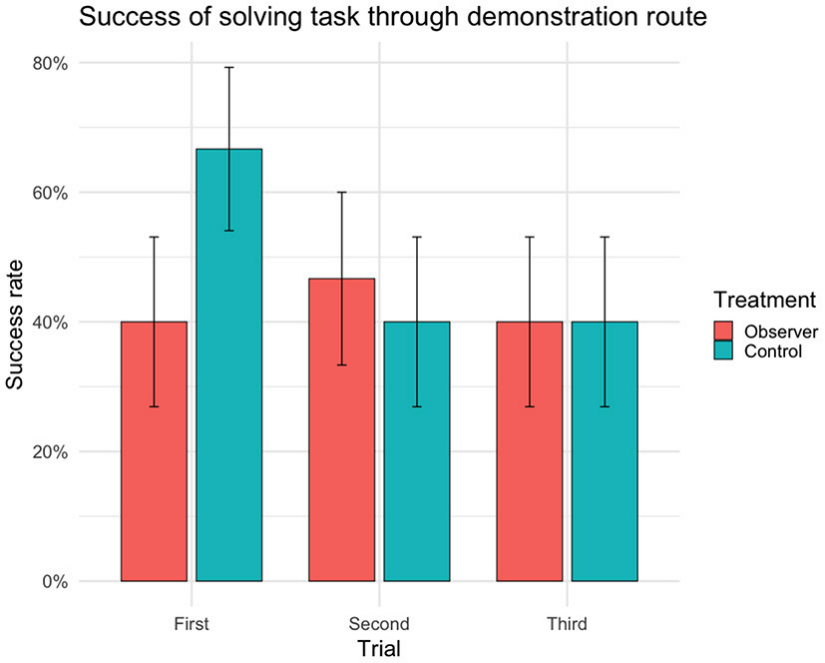
Success rate (sample mean) of reaching the yellow reward bucket and solving task within 90 s through demonstration route (i.e. achieving complete success) for each trial (*n* = 30 cows, 90 trials). Error bars indicating SEM.

There were no significant differences between treatment groups in the success of solving the detour task (Table 1) or in the choice of route (Table 2). In first trial, 27 out of 30 cows (*n* = 14 observers, 13 controls) reached the yellow reward bucket within 90 s regardless of route (Figure 2) and solved the detour task (i.e., achieving complete or partial success). Of these 27 successful cows, 15 individuals (*n* = 6 observers, 9 controls) took the demonstration route (i.e., achieving complete success) in first trial. Although the overall success of solving the task (i.e., regardless of route) was high in first trial, it significantly decreased with the following trials (Figure 2). The fitting of the models showed a significant effect of age on success, with younger cows performing better than older cows in the overall success of solving the task (Table 1) but not in doing so through the demonstrated route (Table 2). This effect of age was insignificant in the *post hoc* analysis of first trial. The *post hoc* analysis further revealed an insignificant effect of treatment (estimate ± SE = 0.35 ± 0.17, *t* = 2.01, *p* = 0.07), suggesting that the controls were more inclined to take the demonstration route, while the observers were more inclined to take the opposite route in first trial (Figure 3).

**Table 1:** Summary of the mixed-effects logistic regression on success of solving detour task regardless of route.

**Variable**	**Levels**	***n***	**EMM**	**SE_(EMM)_**	**Asymp. 95% CI_(EMM)_**	**Df**	***p***	
Age	Continuous	30	1.09	0.38	0.34–1.84	1	0.02	*
Breed	Holstein	19	1.23	0.46	0.33–2.13	1	0.66	ns
	Red	11	0.95	0.54	−0.10–2.00			
Treatment	Observer	15	1.32	0.52	0.30–2.34	1	0.45	ns
	Control	15	0.86	0.46	−0.05–1.76			
Trial	First	30	2.66	0.78	1.13–4.18			
	Second	30	0.66	0.48	−0.29–1.60	2	0.001	**
	Third	30	−0.05	0.45	−0.92–0.83			

Mixed-effects logistic regression done with lme4 package. Variables include age (numeric variable), breed (categorical variable with two levels), treatment (categorical variable with two levels) and trial (categorical variable with three levels). The results are on the logit scale and estimated marginal means have been calculated with emmeans package for each variable: n, number of observations; EMM, Estimated Marginal Mean; SE_(EMM)_, standard error of EMM; Asymp. 95% CI_(EMM)_, asymptotic confidence interval of EMM; Df, degrees of freedom and p-values were calculated in ANOVA with car package.

* *p* < 0.05;

** *p* < 0.01. ns, not significant.

**Table 2:** Summary of the mixed-effects logistic regression on success of solving detour task through use of demonstration route.

**Variable**	**Levels**	**n**	**EMM**	**SE_(EMM)_**	**Asymp. 95% CI_(EMM)_**	**Df**	***p***	
Age	Continuous	30	−0.27	0.35	−0.95–0.41	1	0.13	ns
Breed	Holstein	19	−0.13	0.42	−0.95–0.68	1	0.60	ns
	Red	11	−0.40	0.57	−1.51–0.70			
Treatment	Observer	15	−0.46	0.49	−1.42–0.49	1	0.56	ns
	Control	15	−0.07	0.47	−1.00–0.85			
Trial	First	30	0.16	0.49	−0.80–1.13			
	Second	30	0.39	0.50	−1.36–0.58	2	0.47	ns
	Third	30	−0.58	0.50	−1.56–0.41			

Mixed-effects logistic regression done with lme4 package. Variables include age (numeric variable), breed (categorical variable with two levels), treatment (categorical variable with two levels) and trial (categorical variable with three levels). The results are on the logit scale and estimated marginal means have been calculated with emmeans package for each variable: n, number of observations; EMM, Estimated Marginal Mean; SE_(EMM)_, standard error of EMM; Asymp. 95% CI_(EMM)_, asymptotic confidence interval of EMM; Df, degrees of freedom and *p*-values were calculated in ANOVA with car package.

ns, not significant.

### Latency

**Figure 4:**
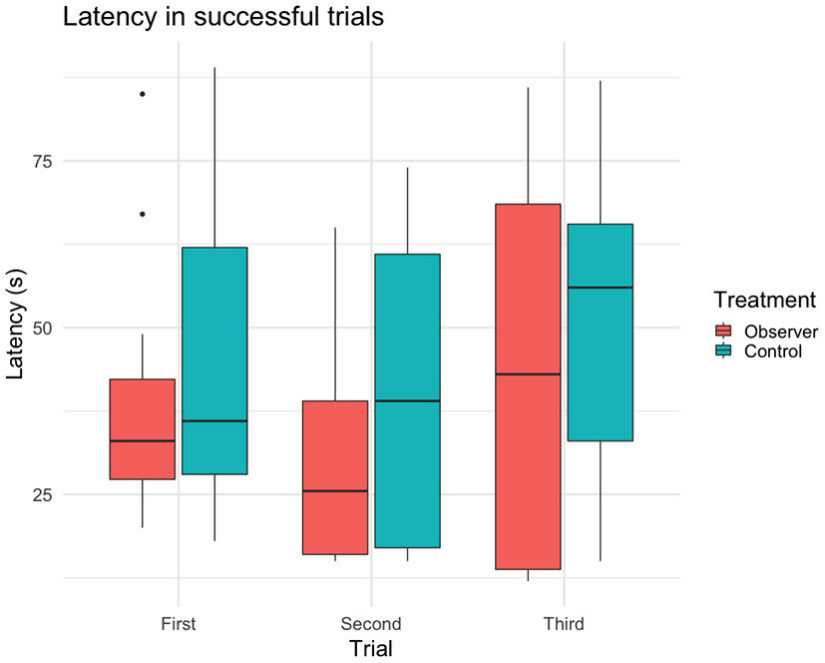
Latency (sample mean) of reaching the yellow reward bucket and solving task in successful trials (*n* = 29 cows, 61 trials).

In 61 out of 90 trials, 29 test cows (*n* = 14 observers, 15 controls) were overall successful in solving the task (i.e., regardless of route) and thus had a latency to reach the reward bucket recorded. In these successful trials, observers had a tendency for shorter latencies than controls (Table 3). Furthermore, there was a significant effect of choice of route on latency, where cows of both treatment groups that took the demonstrated route were significantly faster to reach the reward than cows using the opposite route (Figure 4). Trial had no effect on latency, indicating that cows did not become increasingly faster (or slower) in solving the task over trials (Table 3).

**Table 3:** Summary of the mixed-effects linear regression on latency to reach reward bucket in successful trials.

**Variable**	**Levels**	**n**	**EMM**	**SE_(EMM)_**	**95% CI_(EMM)_**	**Df**	***p***	
Age	Continuous	29	47.1	3.7	39.5–54.8	1	0.97	ns
Breed	Holstein	18	45.7	4.4	36.7–54.7	1	0.69	ns
	Red	11	48.6	5.9	36.4–60.7			
Treatment	Observer	14	41.3	4.9	31.2–51.3	1	0.08	ns
	Control	15	53.0	5.2	42.4–63.7			
Trial	First	27	44.5	4.5	35.6–53.5			
	Second	19	42.3	5.2	31.9–52.8	2	0.15	ns
	Third	15	54.6	5.9	42.7–66.4			
Route	Demonstration	41	39.2	4.2	30.7–47.7	1	0.01	**
	Opposite	20	55.1	5.5	44.0–66.1			

Mixed-effects linear regression done with lme4 package. Variables include age (numeric variable), breed (categorical variable with two levels), treatment (categorical variable with two levels), trial (categorical variable with three levels) and route (categorical variable with two levels). The results are on the response scale and estimated marginal means have been calculated with emmeans package for each variable: n, number of observations; EMM, Estimated Marginal Mean; SE_(EMM)_, standard error of EMM; 95% CI_(EMM)_, confidence interval of EMM; Df, degrees of freedom and p-values were calculated in ANOVA with car package.

** *p* < 0.01. ns, not significant.

### Facing of test arena

All test subjects (*n* = 13 observers, 14 controls) spent time facing the test arena before each trial started. Observers spent a greater percentage of the total time before trial (i.e., during demonstration) facing the test arena (mean ± SD = 45.66 ± 20.10 %) than controls did (mean ± SD = 35.56 ± 17.85 %). The difference between treatment groups was marginally significant (estimate ± SE = 10.4 ± 5.2, *t* = 2.0, *p* = 0.06).

### Concordance

Concordance in route over trials indicated that individual cows of both treatment groups (observers: *n* = 10, mean ± SD = 1.30 ± 0.82, controls: *n* = 10, mean ± SD = 1.50 ± 0.71) were generally consistent in their choice of route to detour. There was no significant difference in concordance between observers and controls (estimate ± SE = 0.60 ± 0.90, *z* = 0.67, *p* = 0.50).

## Discussion

This study aimed to investigate if dairy cows possess the cognitive abilities to acquire new behavior through intra-species social learning in a spatial detour task. It was hypothesized that cows observing a trained demonstrator would be more successful in solving the detour task and do so with shorter latencies compared to control cows. Contrary to what was expected, the results showed no significant differences in success between treatment groups. Instead, age appeared to be the most influencing factor. Moreover, the results showed no effect of treatment on the choice of route, i.e., observer cows did not favor the demonstration route. When only considering trials with successful outcomes (i.e., cows that reached the reward bucket within 90 s regardless of route), the latencies of cows choosing the demonstrated route were significantly shorter than the latencies of those choosing the opposite route, even though the routes provided an equal distance to the reward bucket. Moreover, successful observers had a tendency to be faster than successful controls, which could indicate some effect of treatment on cows learning the route. An alternative explanation could, however, be that the presence of the demonstrator in the demonstrator pen might have negatively affected the latencies of cows choosing the opposite route, on which they walked next to the demonstrator pen. Nonetheless, control cows might have been slower since they relied solely on individual learning mechanisms, which could have affected their latency to solve the detour task.

Individual cows with comparable trial outcomes were generally consistent in their choice of route to detour. This is in contrast to the findings of a lack of consistency in the individual detour behavior of goats (23) and adult sheep (43), but in line with some previous findings of detour behavior in horses (25). Furthermore, observers spent a greater proportion of the time before each trial facing the test arena than controls did, meaning they were likely to have seen the demonstration. Collectively, the results of the study indicate that cows did not utilize social learning mechanisms when solving the applied spatial detour task.

### Motivation

The overall success rate in first trial was high for both observers and controls, with 27 of 30 cows successfully solving the task within 90 s. Surprisingly, for both treatment groups, latency increased over the following trials. This is in contrast with some of the results for horses, who conversely became increasingly faster over trials (25). As latencies increased and cows failed to reach the bucket within 90 s, the previously high success rate decreased. As most cows managed to reach the reward bucket during their first attempt, it seems that the failure to repeat this success in following trials is more likely to be a reflection of a lack of motivation than an inability to solve the task.

Although all cows seemed motivated to obtain food rewards during habituation and training (e.g., consistently seeking out and emptying buckets, as well as fulfilling the habituation criterion), one of the main challenges during the test was keeping both the test subjects and demonstrator cows motivated throughout repeated demonstrations and trials. Several factors could be at play. The test was performed in the hours between morning and afternoon (from 09:00 to 15:00 h) when the cows normally would be out on pasture, grazing and ruminating/resting. Anecdotally, grazing in the test arena increased during the second and third trial (compared to first trial), and cows would graze on all sides of the obstacle before approaching the reward bucket. Furthermore, some cows would successfully detour the obstacle without approaching the bucket and thus rendering the trial unsuccessful. The closer to the afternoon milking, the more difficult it became to drive the cows from the cowshed to the test arena, indicating a strong motivation to remain indoors. The weather during the test days was generally warmer than during training, with temperatures reaching up to 30°C, clear blue skies, and no wind. This is considered very warm for Swedish summer. The heatwave also brought on an increase in both regular flies (*Musca spp*.) and biting giant horseflies (*Tabanidae spp*.), and the insect harassment clearly affected both test subjects and demonstrator cows. To increase motivation, future studies could benefit from using heifers or dry cows placed under a limited feed regime, as opposed to milking cows with access to plentiful amounts of the same type of concentrates as the food reward, or potentially using a higher-value food reward. Testing in an indoor setting could provide a more controlled environment and eliminate grazing opportunities during the test.

### Age

Younger cows were more successful compared to older cows in overall solving the detour task and reaching the yellow reward bucket, however, they were not faster in doing so. Moreover, the effect of age on success does not imply that they were learning from the demonstrator as there was still no effect of treatment on success or the choice of route. Furthermore, as this effect of age was insignificant in first trial where the overall success rate was highest, it is likely a reflection of a decrease in the motivation of older cows during following trials.

Motivation toward the acquisition of novel information can be defined as curiosity (44). In young horses, the motivation to explore novel objects (i.e., the level of curiosity) has been shown to be positively associated with learning performance in tests based on both positive and negative reinforcement (45). More research is needed to determine if younger cows are more curious than older cows, and if so, how curiosity acts to motivate the acquisition of novel information. The effect of age on success in this study further underlines the benefits of using heifers in future studies.

### Demonstration

As mentioned, the dominance relationship between demonstrator and observer has been suggested to be important for the facilitation of social learning (30). This may be because lower-ranking animals are more attentive toward higher-ranking animals to avoid aggression, or because higher-ranking animals display better fitness and therefore are more attractive to learn from (1, 22). Older cows are more likely to be dominant (46), but as suggested by McVey et al. (25), leadership status may also affect demonstrator significance in a social learning context, and different types of leadership may be important depending on the task. In a spatial detour task, the most relevant leader may be the one who can successfully initiate movement of one or more followers. In this herd, the demonstrators stood out as (i) being initiators of movement, i.e., showing leadership (47), (ii) displaying low fear and high curiosity of the experimenters, which was considered crucial for training, and (iii) winning agonistic encounters with other herd members, i.e., indicating a level of dominance (31, 32). However, these cows were only 3 years old in a group where, at the time of testing, age ranged from 2 to 9 years with a mean of 3.7 years. As research shows that age is likely to play a role in dominance (46) and thus also in social learning (1, 30), future studies may benefit from a more thorough investigation into the social hierarchy (32, 48) and different leaders (47) of the herd before selecting demonstrators.

One of the components of actual social learning is goal emulation (6). To ensure that the observer cows solved the detour task with the goal of accessing the food reward, and not simply as a result of seeking social companionship and thus the proximity of the demonstrator cow, the demonstrator was removed from the test arena after each demonstration. However, as isolation has been shown to increase stress and negatively impact learning and performance in cattle (49), the demonstrator was placed in an adjacent demonstrator pen for social buffering. This demonstrator pen was located next to the obstacle (as opposed to next to the starting pen and the observer) due to concern that the test subjects otherwise might be more inclined to remain close to the demonstrator pen than to solve the task. As the presence of a conspecific has shown to impact detour strategy (25), the demonstration route was placed on the opposite side of the obstacle. Thus, avoiding any potential effect of the demonstrator's presence on the observer's choice of route being mistaken for an effect of social learning from the demonstration. This meant that the demonstrations began with the demonstrator walking toward the observer before rounding the obstacle and proceeding in demonstrating the way to the food reward. It seems likely that this might add another layer of complexity to the demonstration, in terms of both visual and olfactory cues, for the observer to interpret. Future studies featuring a similar task should consider a design that allows for a demonstration starting point that is closer to that of the observer.

An ideal demonstration would have included the demonstrator cow performing the demonstrations independently (i.e., without the human experimenter) and in the same manner in each trial and for each observer cow. This was not achievable and, therefore, the human experimenter had to lead the demonstrator (using the black bucket) to ensure conformity between demonstrations. Hence, had the results of this study shown an effect of demonstration that was indicative of social learning, it could have been discussed whether this effect was evidence of intra-species or inter-species social learning. Future research should ideally design detour tasks that allow for conspecific demonstration of the task, and when not possible, movements of the human experimenters, both before, during and in between trials, should be taken into careful consideration.

### Social transmission

Surprisingly, when looking at first trial, the controls appeared to favor the demonstration route, and the observers the route next to the demonstrator pen. Although this effect was insignificant, this raises some questions about the potential role of social transmission. One explanation for the observers favoring this route (while the controls did not) could be that the demonstrator was still chewing on the food from her bowl when the trial started, which may have served as a stimulus enhancement. On the other hand, the demonstrators were observed grazing and chewing on grass throughout the test and in both control and observer trials. Another, perhaps more plausible, explanation could be that watching the demonstrator exit the test arena after each demonstration served as a stimulus enhancement toward the demonstrator pen and potentially the social companionship of the demonstrator cow. This could mean that cows may be equally or more motivated by social companionship than by the food reward used in this study, or it could mean that they interpreted the demonstrator pen as the way out of the test arena and into the pasture.

The difference in choice of route between observers and controls evened out over trials. Regardless, clearer results may be achieved by controlling for social transmission. One alternative could be to implement double control groups; one group where the cows can observe the demonstrator eating from the reward bucket behind the cattle gates before exiting to the demonstrator pen (i.e., partial demonstration), and one group kept as an absolute control (i.e., no demonstration). This would likely need to be compensated with an increase in demonstrators and/or test days, to ensure that the demonstrator cows' motivation doesn't further decrease.

### Practical implications

The results of this study add to the sparse body of knowledge of social learning in livestock ungulates. It further serves as a starting point for future research on the cognitive mechanisms utilized by cows faced with spatial problem-solving in a social context. As all but three cows successfully solved the detour task in the first trial, with a lack of effect of treatment on both the overall success of solving the task and on doing so by use of the demonstration route, it seems plausible that most cows learned how to navigate around the obstacle through individual associative learning.

The lack of evidence of social learning does not necessarily mean that these cognitive mechanisms are absent in cattle. It is possible that social learning is not the primary strategy for acquiring new behavior in this specific situation and that the design of this detour task was too simple for there to be a detectable effect of any secondary strategies. Similar results for horses were found by McVey et al. (25), who pointed to the possibility that social learning might be reserved for when individual learning is ineffective. There is also the possibility that the observers did not fully understand the demonstration and that individual learning thus was employed as a secondary, rather than a primary, strategy for solving the task. A spatial problem-solving task with an increased degree of difficulty (and thus a higher risk of failing through individual learning), and a design that allows for a less complex demonstration, could provide a clearer result of the occurrence of social learning in cattle.

The strategy for acquiring new behavior could also be specific to the situation, meaning that cattle may have the ability to utilize social learning in other situations that are unrelated to spatial problems. As studies have shown that lambs can learn to feed select, and thereby avoid poisonous plants, from grazing with their dams (9, 10), it is possible that such risk-reducing foraging strategies (11) could also be utilized by calves grazing with their dams. However, it is also possible that the adaptive value of social learning, at least between peers, is relatively low for grazing cattle in comparison to predator species with more complex foraging behavior. As such, it may be that simpler processes of information transfer (i.e., social transmission mechanisms) have provided enough evolutionary advantages to cattle through, e.g., social facilitation of synchronized behaviors (50–52), feed locations (13), how to graze (7, 8), and the appropriate response to a frightening stimulus (16).

Learning more about the cognition of cattle is important in several aspects, including cattle welfare and the sustainability of the meat and dairy industry. Assumptions of cattle's ability to emulate or imitate the behavior of others (during e.g., moving of animals to new pastures, loading for transport, etc.) can potentially lead to frustration in livestock handlers (53), and rough handling of cows failing to meet these expectations (54). Such handling has negative welfare implications for the individual cow, with the handling-induced stress and fear also leading to production losses (55), and an increased risk for animal-related injuries to livestock handlers (56–58). As such, a deeper understanding of the cognition of cattle may help in the development of housing systems and management routines and has the potential to improve cattle welfare as well as handler safety, while avoiding unnecessary production losses.

## Conclusion

The results of this study indicate that cows did not rely on social learning mechanisms when solving the applied spatial detour task. Instead, it seems plausible that most cows learned to solve the detour task through individual learning. More research is needed to determine if this was because cattle do not possess the cognitive mechanisms necessary for social learning or if, in this specific situation, cows primarily utilized other strategies for acquiring novel behavior. Designing a detour task with an increased degree of difficulty that also allows for a less complex demonstration may, together with the implementation of a control for social transmission, provide clearer results. As age and motivation appeared to play a role in this study, future studies could benefit from using older demonstrators, younger test subjects, test subjects placed under a limited feed regime, and/or from using a higher-value food reward.

## Data availability statement

The raw data supporting the conclusions of this article will be made available by the authors, without undue reservation.

## Ethics statement

The animal study was reviewed and approved by Board for Animals in Research and Teaching, SLU, Uppsala, Sweden. Written informed consent for participation was not obtained from the owners because oral informed consent was obtained prior to the experiments.

## Author contributions

MR applied for and was later awarded funding for the study. JS brought the idea for the experiment. MR, JS, and JY collaborated on finetuning the test adapted from previous work done by MR. MR, JS, and JY participated in the training, habituation, and preparations of the experiment. JS and JY executed the experiment. MR and JS were in charge of data retrieval and editing and performed the statistical analysis together. JY and HB contributed to the interpretation of the results. JS wrote the first draft of the paper under supervision of MR. All authors provided critical feedback and contributed to proof reading and fine-tuning the paper for publication.

## Funding

The authors would like to thank our funders Graméns Fund, Alnarp, Sweden and the Swedish University of Agricultural Sciences.

## Conflict of interest

The authors declare that the research was conducted in the absence of any commercial or financial relationships that could be construed as a potential conflict of interest.

## Publisher's note

All claims expressed in this article are solely those of the authors and do not necessarily represent those of their affiliated organizations, or those of the publisher, the editors and the reviewers. Any product that may be evaluated in this article, or claim that may be made by its manufacturer, is not guaranteed or endorsed by the publisher.
